# Mutation detection using ENDO1: Application to disease diagnostics in humans and TILLING and Eco-TILLING in plants

**DOI:** 10.1186/1471-2199-9-42

**Published:** 2008-04-23

**Authors:** Karine Triques, Elodie Piednoir, Marion Dalmais, Julien Schmidt, Christine Le Signor, Mark Sharkey, Michel Caboche, Bénédicte Sturbois, Abdelhafid Bendahmane

**Affiliations:** 1 Unité Mixte de Recherche en Génomique Végétale, 2 rue Gaston Crémieux, CP5708, 91 057 Evry Cedex, France; 2 University of Massachusetts Medical School, Worcester, Massachusetts, USA; 3 Unité Mixte de Recherche en Génétique et Ecophysiologie des Légumineuses, Domaine d'Epoisses, 21110 Bretenières, France

## Abstract

**Background:**

Most enzymatic mutation detection methods are based on the cleavage of heteroduplex DNA by a mismatch-specific endonuclease at mismatch sites and the analysis of the digestion product on a DNA sequencer. Important limitations of these methods are the availability of a mismatch-specific endonuclease, their sensitivity in detecting one allele in pool of DNA, the cost of the analysis and the ease by which the technique could be implemented in a standard molecular biology laboratory.

**Results:**

The co-agroinfiltration of ENDO1 and p19 constructs into *N. benthamiana *leaves allowed high level of transient expression of a mismatch-specific and sensitive endonuclease, ENDO1 from *Arabidopsis thaliana*. We demonstrate the broad range of uses of the produced enzyme in detection of mutations. In human, we report the diagnosis of the G1691A mutation in *Leiden factor-V *gene associated with venous thrombosis and the fingerprinting of HIV-1 quasispecies in patients subjected to antiretroviral treatments. In plants, we report the use of ENDO1 system for detection of mutant alleles of *Retinoblastoma*-*related *gene by TILLING in *Pisum sativum *and discovery of natural sequence variations by Eco-TILLING in *Arabidopsis thaliana*.

**Conclusion:**

We introduce a cost-effective tool based on a simplified purification protocol of a mismatch-specific and sensitive endonuclease, ENDO1. Especially, we report the successful applications of ENDO1 in mutation diagnostics in humans, fingerprinting of complex population of viruses, and in TILLING and Eco-TILLING in plants.

## Background

Scanning DNA sequences for mutations and polymorphisms is an analytic tool in a broad range of disciplines. However, identification of such mutations and polymorphisms in long stretches of DNA and in large numbers of samples by direct sequencing is not a trivial exercise. Several mutation detection techniques based on the physical properties of single stranded DNA or heteroduplex DNA [1-5] have been described. Among such methods are conformation sensitive gel electrophoresis (CSGE) [3], denaturing gradient gel electrophoresis (DGGE) [6], constant denaturing capillary electrophoresis, (CDCE) [7], Temperature Gradient Capillary Electrophoresis (TGCE) [8], single strand conformation polymorphism (SSCP) [2] and denaturing high-performance liquid chromatography (DHPLC) [1]. Other methods exploit chemicals like groove binders or chemicals that cleave single strand DNA at the mismatch site in heteroduplex DNA [9].

Single strand specific nucleases have also been used to cleave heteroduplex DNA at the mismatch site [10-13]. Among them, CEL I is a mismatch specific endonuclease [14] that is widely used for reverse genetics in plants, animals and bacteria [15-22] and for disease diagnostic in human such as cancers related to *BRCA1 *[23,24]. Enzymatic mutation detection is advantageous over other popular mismatch detection systems, like TGCE and DHPLC [8,25-27] because mismatch specific endonucleases allow to screen large stretches of DNA without reducing diagnostic sensitivity or specificity, while at the same time providing information about the location of the mutation. However, many mismatch specific cleavage enzymes were reported to cleave preferentially certain types of mismatches, display low sensitivity or lead to a high background [10,11,20,28,29].

Previously, we reported the biochemical analysis of five S1 type nucleases from *Arabidopsis thaliana*. We demonstrated that one of them, ENDO1, is a mismatch specific endonuclease, which cleaves with a high efficiency all types of mismatches and has a high sensitivity, detecting one allele in pool of sixty [30]. Here, we report a very simple protocol for the expression and the preparation of ENDO1. Especially, we report the use of ENDO1 in combination with universal labelled primers as a cost-effective tool for mutation diagnostics in humans, plants and viruses.

## Results

### Rapid preparation of ENDO1

**Figure 1:**
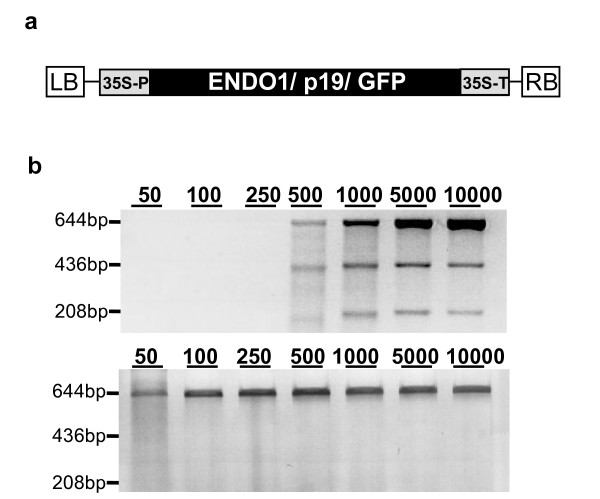
**Expression and analysis of ENDO1 activity**. (a) Construction bearing, ENDO1, TBSV p19 or GFP ORFs. The box indicates the ORF of the protein to be expressed, cloned between the 35S promoter (35S-P) and the transcriptional terminator (35S-T) of CaMV. LB and RB indicate the left and right border of the T-DNA respectively. (b) Heteroduplex DNA was created based on two clones that differ in single base insertion at position 436 bp of the 644 bp test PCR product. Heteroduplex DNA was digested with the tobacco protein extract derived from leaves co-agroinfiltrated with ENDO1 and TBSV p19 constructs (upper picture) after 50, 100, 250, 500, 1000, 5000 and 10000-fold dilution and analyzed on 1.5% standard agarose gel. Protein extract from leaves co-agroinfiltrated with TBSV p19 and GFP, instead of ENDO1 construct, was used as a negative control (lower picture).

ENDO1 is expressed at low level in *Arabidopsis *during plant senescence [31]. In order to facilitate the expression of the protein, the ENDO1 ORF was inserted between the 35S promoter and the transcriptional terminator of CaMV in the binary vector pBIN61 (Figure 1a) [30]. The construct was transformed into *Agrobacterium *and co-agroinfiltrated into *N. benthamiana *leaves in the presence of the p19 protein of tomato bushy stunt virus (TBSV), that prevents the onset of post-transcriptional gene silencing (PTGS) in infiltrated tissues and allows a high level of transient expression (Figure 1a) [32]. As negative control the plants were also agroinfiltrated with a construct expressing the green fluorescent protein (GFP).

Ten grams of agroinfiltrated leaves were homogenized and cleared by centrifugation in the absence and in the presence of 30% ammonium sulphate. Ammonium sulphate was then added to 80% final concentration in the supernatant and the pelleted proteins were resuspended in buffer containing 50% glycerol, dialysed against the same buffer and stored at -80°C.

To test whether ENDO1 obtained from this simplified purification protocol is active we tested the protein extract on heteroduplex DNA created from two clones that differ by a single base insertion (Figure 1b). In this experiment we predicted that if the ENDO1 preparation contains a mismatch specific endonuclease, the heteroduplex DNA will be cleaved at the mismatch site releasing two bands of 208 bp and 436 bp (Figure 1b). Duplex DNAs were incubated with dilutions of ENDO1 or GFP protein extracts. ENDO1 led to the predicted digestion product and as expected the GFP control showed undetectable mismatch specific cleavage activity (Figure 1b, lower picture). As shown for His-tag purified ENDO1 [30] and CEL I [20], at high concentration (50 to 250 fold dilution, Figure 1b), ENDO1 presented double strand DNAse activity and digested the entire fragments. From this biochemical analysis we concluded that ENDO1 simplified purification led to an active enzyme and the test mutation was detected at dilutions ranging from 500 to 10 000 fold (Figure 1b, upper picture). Based on this analysis and duplicates of this experiment (data not shown), we estimate that the amount of enzyme produced from 10 grams of agroinfiltrated leaf material, and used at 10 000 fold dilution, is enough to carry more than a million mismatch detection tests.

### Detection of G1691A mutation in factor V

**Figure 2:**
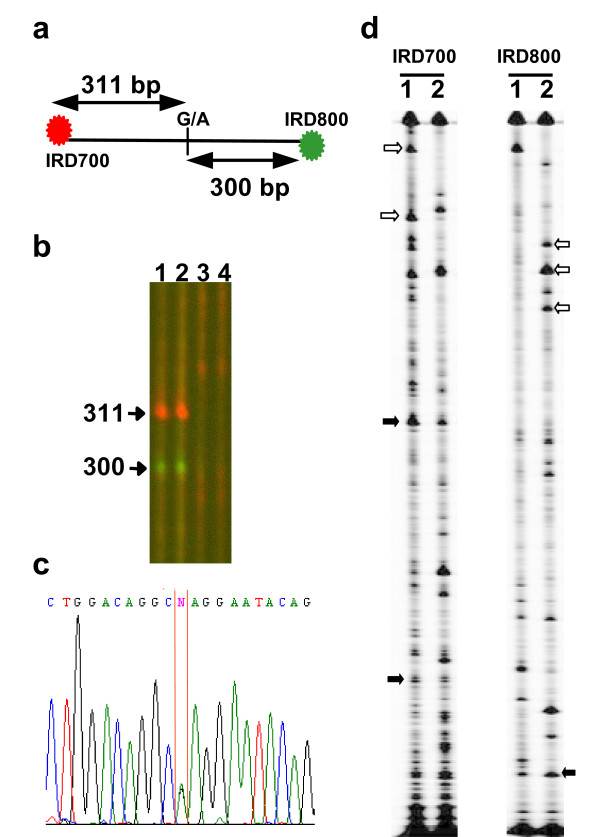
**Detection of G1691A mutation in factor V in human and Fingerprinting of HIV-1 quasispecies**. (a) Schematic representation of 611 bp DNA fragment of factor V indicating G1691A mutation associated with venous thrombosis. The red and green dots indicate the position of IRD700 and IRD800-labelled primers, respectively. (b) Genomic DNA was extracted from leg hairs of a non-carrying (lane 3 and 4) and heterozygous individual for the G1691A mutation (lane 1 and 2). The 611 bp DNA fragment corresponding to the region harboring the mutation was PCR amplified, denaturated/renaturated prior to digestion with ENDO1 and analyzed on on LI-COR4300 DNA Analyzer. Sizes of the digested fragments are indicated on the left. (c) Chromatogram of the sequence of the human Factor V fragment from heterozygous individual showing the G1691A mutation. (d) Genomic DNAs were extracted from peripheral blood mononuclear cells of an HIV-1 infected patient before (lane 1) and after 48 weeks of antiretroviral therapy (lane 2). DNA fragment of 843 bp coding for the reverse transcriptase gene of HIV-1 was PCR amplified, denaturated/renaturated prior to digestion with ENDO1 and analyzed on LI-COR4300 DNA Analyzer. The collected image from both channels is shown (IRD700 and IRD800 channels). Examples of bands of invariant intensity that indicate stable mutations are indicated by filled arrow. Examples of bands indicating mutations that either appeared or were lost upon treatment are indicated by empty arrows.

We tested ENDO1 prepared as above in mutation diagnostics in humans. As a proof of concept, we investigated the detection of the factor V G1691A mutation associated with venous thrombosis [33,34]. Genomic DNA was extracted from individuals harboring or not the G1691A mutation and analyzed using ENDO1 system. To reduce the diagnostic cost, we combined the ENDO1 mismatch detection system with the use of labelled universal primers in the amplification of the target sequence (Table 1). In this analysis we expected that if the 611 bp heteroduplex DNA fragment of factor V is cleaved at the mismatch site, two bands of 300 bp and 311 bp would be released (Figure 2a). Results obtained from both 700 and 800 channels of the LI-COR4300 show that ENDO1 recognizes the G1691A mutation (Figure 2b, lane 1 and 2). No cleavage was observed on PCR product obtained from a control person not carrying the G1691A mutation (Figure 2b, lane 3 and 4). The experiment was repeated three times with similar results (data not shown). As expected the presence of G1691A mutation was confirmed by sequencing in the patient heterozygote for the mutation (Figure 2c). Because mutation detection relies on heteroduplex formation between two alleles, detection of the G1691A mutation at the homozygous state will require mixing genomic DNA from a control individual not harbouring the mutation with that of an individual to be tested.

**Table 1:** Primers used to amplify target loci

**Targets**	**Primer name**	**Primer sequence**
Factor V	M13F-FV Forward	**cacgacgttgtaaaacgac **ttaattggttccagcgaaagc
	M13R-FV Reverse	**ggataacaatttcacacagg **tccaataccaacagacctgg
Reverse Trans.	M13F-RT Forward	**cacgacgttgtaaaacgac **taggacctacacctgtcaacataa
	M13R-RT Reverse	**ggataacaatttcacacagg **cttctgtatgtcattgacagtcca
RBR exon10-11	RB-1N1 Forward	ggagcagtcagaataagcggaatg
	RB-1N1 Reverse	taaccaagcccacatcgacgtcatc
	RB-1N2 Forward	**cacgacgttgtaaaacgac **ggctacaattatctcagcagataag
	RB-1N2 Reverse	**ggataacaatttcacacagg **gtcctcttactttccggttgctgaa
RBR exon14-17	RB-2N1 Forward	tgaagaagacgccctgtttggcatc
	RB-2N1 Reverse	gttgcttaagtgaagaagccgatct
	RB-2N2 Forward	**cacgacgttgtaaaacgac **tgagtgtcttggaaaatgattacga
	RB-2N2 Reverse	**ggataacaatttcacacagg **aggcaaggaaggcacaggaggcaat
BAC F15K9	Tail-At Forward	**cacgacgttgtaaaacgac **gctgattatgggatgatgatatt
	Tail-At Reverse	**ggataacaatttcacacagg **aaaaagcagcgagttataggtttac

Targets: corresponding GenBank accession numbers are Human coagulation factor V (Factor V, Z99573), HIV-1 Reverse Transcriptase (Reverse Trans, AF033819), *Pisum sativum *Retinoblastoma-related gene RBR (AB012024) and *Arabidopsis thaliana *BAC F15K9 sequence (AC005278).

Primer sequence: in bold are indicated M13 forward and reverse universal tails; in simple text is indicated the part of the primer specific to the target sequence.

### Fingerprinting of HIV-1 quasispecies

Human immunodeficiency virus type 1 (HIV-1) present in infected individuals has been described as quasispecies of related but distinct viruses [35-38]. When the selective pressure of antiretroviral therapy is exerted on such a population, drug-resistant mutants may emerge and consequently lead to treatment failure [35,37]. The objective of this work was to assess the HIV-1 quasispecies evolution during different treatments. Direct sequencing of PCR amplified HIV-1 DNA fragment from an infected individual will detect only predominant mutations. Detailed analysis of the HIV quasispecies using sequencing would require the cloning of PCR products and systematic sequencing of individual clones. A list of alternative technologies have been also tested to identify minor HIV drug-resistant populations [39-43].

Previously we demonstrated that ENDO1 can detect rare alleles in pools of DNA [30]. Thus, we tested the use of ENDO1 system as a fingerprinting protocol to assess the HIV-1 quasispecies evolution during different treatments. We focused the analysis on the sequence variation in a DNA fragment of 843 bp coding for the reverse transcriptase gene of HIV-1. Genomic DNAs were extracted from peripheral blood mononuclear cells of an HIV-1 infected patient before (Figure 2d, lane 1) and after 48 weeks of antiretroviral therapy (Figure 2d, lane 2). The reverse transcriptase gene was PCR amplified and analysed using ENDO1. Two different patterns of bands, representing fingerprints of HIV-1 quasispecies at each time, were observed (Figure 2d). Comparing the overall patterns and changes in intensity of particular fragments allowed the identification of mutations that either appeared or were lost upon treatment (Figure 2d, empty arrows). Bands of invariant intensity indicate stable mutations (Figure 2d, filled arrows).

### Exploitation of ENDO1 in TILLING

TILLING (Targeting Induced Local Lesions IN Genomes) method combines the induction of a high number of random mutations with mutagens like Ethyl Methane Sulfonate (EMS) and mutational screening systems to discover induced mutations in sequence DNA targets [44]. This reverse genetic strategy encompasses all types of organisms as plants, animals, bacteria, without being subjected to the genome size [15-19,45,46].

**Figure 3:**
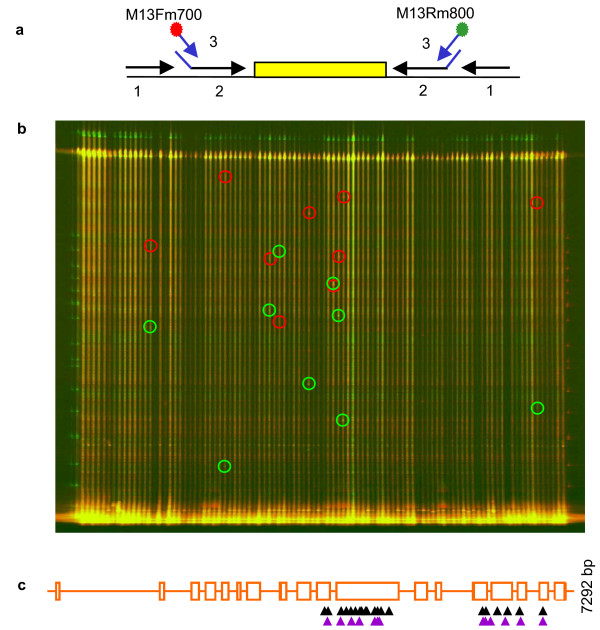
**TILLING using ENDO1**. (a) Universal primers Tilling strategy. Targets were amplified by PCR with gene specific primers (black arrows 1), followed by a nested-PCR with internal gene specific (black parts of arrows 2) primers containing a universal M13 tail (blue parts of arrows 2), in combination with IR DYE-labeled universal M13 primers (blue arrows 3); the red and green dots indicate the position of IRD700 and IRD800-labelling, respectively. The sequence of the primers are indicated in Table 1 (b) Example of a *RBR *Tilling screen on 8-fold pooled pea DNA. The gel images represent the superposed image from the IRD700 and IRD800 channels. The sizes of the cleavage products (circled) from the 2 dyes-labeled DNA strands (red or green) add up to the size of the full-length PCR products (top of the gel). PCR artifacts are distinguishable from true mutants by yellow points (red and green added) as they appear of the same size in both channels. The size of the cleavage product (sizing ladder in the first and last lanes) indicates approximately where the SNP is located in the fragment. (c) Graphic representation of mutations identified in two regions of *RBR *gene. This drawing was made using the PARSESNP program [54], which maps the mutation on a gene model to illustrate the distribution of mutations. The purple and black triangles represent silent and missense mutations, respectively. The black lanes indicate the two tilled amplicons.

To test whether ENDO1, purified using the simplified protocol, in combination with universal primers is efficient in TILLING, we screened 4717 M2 families of *Pisum sativum*-EMS mutagenized population (Dalmais et al., unpublished data) for induced mutations in the *Retinoblastoma-Related *gene, *RBR*. The RBR protein is well-known as a regulator of cell division, differentiation and endoreduplication in plants [47]. So far, the use of insertion mutants to investigate the roles of RBR in plant development has been restricted, due to the gametophytic lethality [48]. Two DNA fragments of 1078 bp and 972 bp of RBR were PCR amplified using nested PCR in combination with universal primers (Table 1; Figure 3a). PCR products were digested with ENDO1 and analysed on LI-COR4300 gels as described above (Figure 3b). From 1078 bp and 972 bp amplicons we obtained 34 and 16 mutants, respectively (Figure 3c). From this, 3 were intronic, 17 were silent and 30 were missense mutations. No nonsense mutants were recovered, likely because of their gametophytic deleterious effects [48].

To evaluate the robustness of ENDO1 purified using the simplified protocol, in combination with universal primers for mutation detection, *RBR *TILLING was carried out with conventional method based on Ni-Column-purified ENDO1 and gene specific primers. A similar number of mutants were identified (data not shown). Based on the TILLING of *RBR *and other targets (data not shown) we concluded that the ENDO1 simplified protocol is suitable for high throughput TILLING screen.

### Exploitation of ENDO1 in Eco-TILLING

EcoTILLING is a means to determine the extent of natural sequence variation across many germplasms, enabling both SNP discovery and haplotyping [22]. This technique is now applied to rice, maize, lotus, poplar [49], *Brassica*, zebrafish, *Drosophila*, *Caenorhabditis *and human [50], indicating its broad applicability.

**Figure 4:**
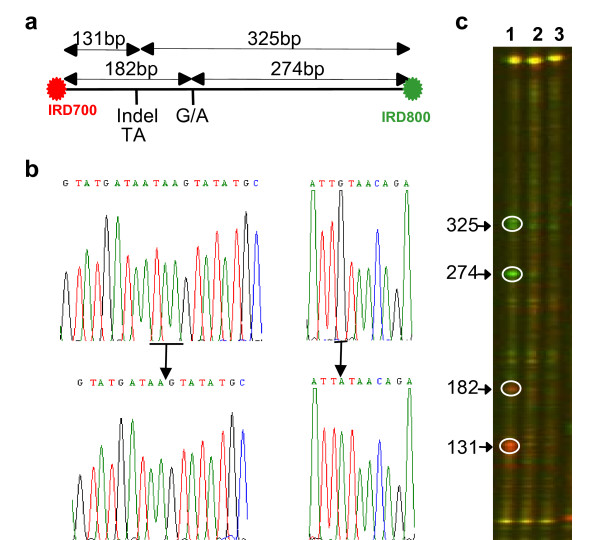
**Eco-TILLING using ENDO1**. (a) Schematic representation of a 456 bp genomic DNA fragment of *Arabidopsis thaliana *showing two sequence polymorphisms, 3-base INDEL and the G to A substitution, between Columbia and Landsberg ecospecies.(b) The 456 DNA fragment was PCR amplified using the primers indicated in Table 1. Heteroduplex DNA is prepared by mixing equimolar amounts of the two PCR products, generated from Columbia and Landsberg ecospecies, heating to denature the DNA followed by cooling to renature it. Lane 1 indicates heteroduplex DNA. Lane 2 and 3 indicate homoduplex DNA generated from Columbia and Landsberg ecospecies, respectively. Homo and heteroduplex DNAs were digested with ENDO1 and analyzed on LI-COR4300 DNA analyzer. Expected band sizes are indicated on the left of the panel. (c) DNA sequencing chromatograms surrounding the 3-base INDEL (Left panel and underlined) and the G to A substitution (Right panel and underlined) between Columbia (upper sequence) and Landsberg (lower sequence) ecospecies.

In *Arabidopsis thaliana*, we analysed natural sequence variations within a DNA fragment of 456 bp (Figure 4a) using the ENDO1 system (Figure 4c) and sequence analysis (Figure 4b), in two *Arabidopsis *ecotypes, Columbia and Landsberg. We expected that if the 456 bp heteroduplex DNA fragment, created from Columbia and Landsberg pooled genomic DNAs (lane 1), is cleaved at the mismatch sites, four bands of 325, 274, 182 and 131 bp would be released. Results obtained from both channels 700 and 800 of the LI-COR4300 show that ENDO1 recognizes, with a high specificity the three base pair INDEL and the G to A substitution (Figure 4c, lane 1). No cleavage was observed on PCR product obtained from the control Columbia and Landsberg-homoduplex DNAs (Figure 4c, lane 2 and 3, respectively). The obtaining of four bands indicates that ENDO1 cleaves the DNA fragments partially. This characteristic of ENDO1 enhances the potential of the system, because it is able to detect sequence variation in a DNA fragment carrying multiple mutations in a single reaction.

## Discussion

Mutation detection is often an expensive and time-consuming obstacle to many molecular genetic applications including reverse genetics and clinical diagnostics. In this work we describe an enzymatic mutation detection technique based on ENDO1. We focused our work on the main limitation of any enzymatic mutation detection system, the production of a mismatch specific endonuclease. CEL I is the most commonly used endonuclease in TILLING and EcoTILLING in plants and animals [15-19,21,22,45]. Three protocols are reported for the purification of CEL I from celery stalks. The highly purified enzyme is prepared from 105 kg of celery stalks and a more simplified protocol use 7 kg. Both protocols use a series of chromatography purifications steps requiring special equipments and skills in biochemistry. A more simplified protocol was reported [20] that use 0.5 kg of store-bought celery stalks. However, the minimum information on the name of the celery variety and the age of the plants to be used in the purification are missing, which renders the quality of produced enzyme uneven.

In this work, the co-agroinfiltration of ENDO1 and p19 constructs into *N. benthamiana *leaves allowed high level of transient expression. ENDO1 represented at least 6% of total proteins (data not shown) and from 10 grams of plant material, we routinely obtain enough enzyme to carry out more than a millions mismatch detection tests. The crude purified ENDO1 was found to be stable, allowing at least 4 months storage at -20°C. For longer period storage we recommend to purify His-Tagged ENDO1 on Ni-NTA agarose beads as described previously [20]. The His-tag purified ENDO1 was found to be very stable, allowing at least two years storage at -80°C, one year storage at -20°C and more than four months storage at 4°C.

Several new techniques of mutation scanning based on microchips and/or sequencing or re-sequencing of a given genome are currently being developed [51]; however, these technologies yet have in common the major drawback of their cost. Such tools, despite their outstanding potential for mutation scanning, are now likely to be restricted for such applications to big laboratories specialised in sequencing or genotyping. On the other hand, using ENDO1 system, we demonstrated its universality as a low-cost strategy for easy high-throughput diagnostic of genetic mutations in different genomes, including those incompletely sequenced like pea. In humans, the ENDO1 system permitted the diagnosis of the well-known genetic mutation, G1691A in Factor V gene, which is often associated with activated protein C resistance that is a common risk factor for venous thrombosis [33]. In viruses, we described a fingerprinting protocol to assess the HIV-1 quasispecies evolution during different treatments. We showed that the detection of quasispecies using universal primers with two PCR-rounds or using specific primers in one PCR-round gave the same results (data not shown). The natural progression of viral species within HIV-1 infected patients and/or the correlation of the appearance of quasispecies with the resistance to certain antiretroviral could be essential for clinical survey. In plants, we demonstrated the use of ENDO1 in combination with universal primers to decrease further the cost of the screening in TILLING and Eco-TILLING, two strategies that require high throughput screening protocols.

The *Agrobacterium *transient-expression assay could be used not only to produce (at low cost) large amounts of ENDO1, but also to over-express other nucleases for which the expression in *bacteria *is toxic or require post-translational modifications. Initial attempts to express ENDO1 and CEL I in *E. coli *were unsuccessful and when we succeeded, the expressed proteins showed no DNase activity (data not shown).

## Conclusion

The ENDO1 preparation protocol described in this work does not require any particular equipment or skills and could be implemented in any standard molecular biology research laboratory. The produced ENDO1 was successfully used in mutations diagnostics in different genomes. In humans, the ENDO1 system permitted the diagnosis of the well-known genetic mutation in Factor V gene, which is often associated with venous thrombosis. In viruses, we described a fingerprinting protocol to assess the HIV-1 quasispecies evolution during different treatments. In plants, we demonstrated the use of ENDO1 in combination with universal primers to decrease further the cost of TILLING and Eco-TILLING.

## Methods

### Overexpression of ENDO1

pBIN35S-ENDOI construct was described previously [52]. *Agrobacterium *transient overexpression in *Nicotiana benthamiana *was performed as described previously [53] except that the cells were co-infiltrated into *N. benthamiana *leaves in presence of *Agrobacterium *harbouring pBIN61-p19 construct [32]. Agroinfiltrated plants were incubated for at least 60 to 96 hours in the green house before protein extraction.

### Purification of ENDO1

Ten grams of agroinfiltrated leaves were homogenized in 10 ml buffer containing 0.1 M Tris-HCl pH 8, 200 μM phenylmethylsulphonyl fluoride (PMSF), 0.125 mM β mercaptoethanol and 10% of glycerol and cleared by centrifugation at 3,000 × g for 30 min. Ammonium sulphate was added to the supernatant to the final concentration of 30% and the samples incubated on ice for 1 hour were centrifuged at 30,000 × g for 30 min. Ammonium sulphate was added to the supernatant to 80% final concentration and the proteins were precipitated by centrifugation as above. The pellet was resuspended in 500 μl of dilution buffer (50 mM Tris-HCl pH 8, Glycerol 50%, 100 μM PMSF), dialysed against the same buffer and stored at -80°C.

### PCR amplification and mutation detection

In the TILLING screen, target loci were PCR-amplified using nested-PCR and universal primers. The first PCR amplification is a standard PCR reaction using target-specific primers [28]. One microlitre of the first PCR served as a template for the second nested PCR amplification, using a mix of gene-specific inner primers carrying a universal M13 tail (Table 1), in combination with M13 universal primers, M13F700 (CACGACGTTGTAAAACGAC) and M13R800 (GGATAACAATTTCACACAGG), labelled at the 5'end with infra-red dyes IRD700 and IRD800 (LI-COR^®^, Lincoln, Nebraska, USA), respectively. This PCR was carried out with each primers at 0.1 μM, using the following 2 steps cycling program: 94°C for 2 min, 10 cycles at 94°C for 15 sec, primers specific annealing temperature for 30 sec and 72°C for 1 min, followed by 25 cycles at 94°C for 15 sec, 50°C for 30 sec and 72°C for 1 min, then a final extension of 5 min at 72°C. The PCR amplification of factor V, HIV-1 reverse transcriptase and *Arabidopsis *BAC 15K9 DNA fragment was carried out using only the second PCR conditions described above using 30 ng of genomic DNA as template. Mutation detection, in non-purified PCR products, was carried out as described previously [30] except that the crude extracted enzyme was used at 10 000 fold dilution and 0.6 μl of ENDO1-digestion products were loaded on the gel using disposable paper membrane combs (The Gel Company, San Francisco, USA).

## Authors' contributions

KT developed ENDO1 preparation, performed factor V mutation detection and Eco-TILLING. EP and BS set up ENDO1 extraction. MD optimized TILLING protocol. JS carried out TILLING of *RBR*. CLS maintained *Pisum *mutant lines. MS with KT completed HIV-1 quasispecies fingerprinting. MC and AB supervised the study. The manuscript was written by KT, JS and AB.
